# A Cross-Metabolomic Approach Shows that Wheat Interferes with Fluorescent *Pseudomonas* Physiology through Its Root Metabolites

**DOI:** 10.3390/metabo11020084

**Published:** 2021-01-31

**Authors:** Laura Rieusset, Marjolaine Rey, Florence Gerin, Florence Wisniewski-Dyé, Claire Prigent-Combaret, Gilles Comte

**Affiliations:** Ecologie Microbienne, Université Claude Bernard Lyon1, Université de Lyon, CNRS UMR-5557, INRAe UMR-1418, VetAgroSup, 43 Boulevard du 11 novembre 1918, 69622 Villeurbanne, France; laura.rieusset@hotmail.fr (L.R.); marjolaine.rey@univ-lyon1.fr (M.R.); florence.gerin@univ-lyon1.fr (F.G.); florence.wisniewski-dye@univ-lyon1.fr (F.W.-D.); claire.prigent-combaret@univ-lyon1.fr (C.P.-C.)

**Keywords:** *Pseudomonas*, wheat, secondary metabolites, plant-bacteria interaction, metabolomic, signaling, rhizosphere

## Abstract

Roots contain a wide variety of secondary metabolites. Some of them are exudated in the rhizosphere, where they are able to attract and/or control a large diversity of microbial species. In return, the rhizomicrobiota can promote plant health and development. Some rhizobacteria belonging to the *Pseudomonas* genus are known to produce a wide diversity of secondary metabolites that can exert a biological activity on the host plant and on other soil microorganisms. Nevertheless, the impact of the host plant on the production of bioactive metabolites by *Pseudomonas* is still poorly understood. To characterize the impact of plants on the secondary metabolism of *Pseudomonas*, a cross-metabolomic approach has been developed. Five different fluorescent *Pseudomonas* strains were thus cultivated in the presence of a low concentration of wheat root extracts recovered from three wheat genotypes. Analysis of our metabolomic workflow revealed that the production of several *Pseudomonas* secondary metabolites was significantly modulated when bacteria were cultivated with root extracts, including metabolites involved in plant-beneficial properties.

## 1. Introduction

Plant roots contain a large range of metabolites, both primary (i.e., organic acids, carbohydrates and amino acids) and secondary (i.e., alkaloids, terpenoids and phenolic derivatives) [1]. The metabolic content of the roots is dependent on the plant species, but also on plant genotypes, as well as on biotic or abiotic interactions occurring in the rhizosphere [2,3,4]. Some of these metabolites are secreted into the surrounding soil [5,6]. Through exudation of these compounds, roots interact with a wide range of microorganisms to form the rhizosphere microbial community [7]. Shaping of the so-called rhizomicrobiota is carried out by trophic interactions due to available carbon and nitrogen. In addition to being engaged in trophic relationships, low molecular weight compounds can act as signals that trigger biological responses in microorganisms, and thus affect the rhizomicrobiota [1,8,9,10]. Some plant secondary metabolites like coumarin or benzoxazinoids can, for example, influence the composition of the root microbiome [6,8,10].

Among this microbiota, some bacterial strains called plant-growth-promoting rhizobacteria (PGPR) exert beneficial effects on plant growth, development and/or health [11]. It has been demonstrated that different plant genotypes do not interact in the same way with the same potentially plant-beneficial bacteria [10,12,13,14,15,16]. In addition to the selection of specific plant-beneficial strains, assessing the impact of the plant on physiological functions of PGPRs appears of great concern [1]. Although signaling is well described in mutualistic interactions between *Rhizobia* and leguminous plants [4] or between *Frankia* and *Alnus* [17], the signaling effects of plant root metabolites on the physiology of PGPR and on the metabolites they may release in the surrounding medium are still poorly documented [18,19]. However, since biotic interactions in the rhizosphere are mediated by secondary metabolites, investigation of how plants may interfere with PGPR secondary metabolism is a critical issue. Recently, some studies have analyzed the co-regulation of plant and bacterial genes during the interaction of inoculated PGPR or endogenous bacterial endophytes using transcriptomic approaches [18,19,20]. These studies allow the modeling of interaction networks at a functional level, but the influence of each partner on the production of secondary metabolites is not yet clearly known.

In this work, we developed a cross-metabolomic analysis to evaluate the impact of plant root secondary metabolites on the physiology and secondary metabolism of five bacterial strains. This was conducted with three bread wheat genotypes and five *Pseudomonas* strains. Wheat accessions used in this work were Bordeaux 113 and Adular, which are close to each other as they belong to the same Western European group (haplotype X); and Soissons, which belongs to another Eastern European and Mediterranean group (haplotype II) [21]. *Pseudomonas* strains considered in this study were *P. koreensis* JV222, *P. chlororaphis* JV395B and *P. chlororaphis* JV497 isolated from soil or maize roots, as well as *P. kilonensis* F113 and *P. protegens* CHA0 isolated from sugarbeet and tobacco rhizospheres, respectively [14,22,23]. PGPR belonging to the *Pseudomonas* genus are well-studied rhizosphere strains [12,14], interacting with plants and microbial communities due to the biosynthesis and release of bioactive secondary metabolites [24,25]. Recently, a work from our group allowed us to classify fluorescent *Pseudomonas* species in two different groups, i.e., the CPC (*P. corrugata/P. protegens/P. chlororaphis*) clade, which contains species with many plant-beneficial properties; and the FMJK clade (*P. fluorescens/P. mandelii/P. jessenii/P. koreensis*), in which species present few plant-beneficial properties) [14]. Accordingly, strains *P. kilonensis* F113, *P. protegens* CHA0, *P. chlororaphis* JV395B and *P. chlororaphis* JV497 belong to the CPC clade, whereas *P. koreensis* JV222 belongs to the FMJK clade [14]. They produce a wide range of antimicrobial compounds like 2,4-diacetylphloroglucinol (DAPG), pyoluteorin, phenazine or pyrrolnitrin involved in plant protection, as well as indolic derivatives and signaling compounds involved in plant-growth stimulation and plant-bacteria interaction [23,24,25,26,27,28].

Our wheat-*Pseudomonas* metabolomic study highlighted that wheat root extracts from the three genotypes contained numerous detected metabolites, several of which are differentially produced depending on the wheat genotypes. Moreover, we showed that the root extracts of all genotypes strongly influenced the secondary metabolism of all *Pseudomonas* strains, and that there was a complex modulation of bacterial secondary metabolism according to the combination of plant genotypes x strains.

## 2. Results

### 2.1. Characterization of Root Extracts from Wheat Genotypes

**Figure 1:**
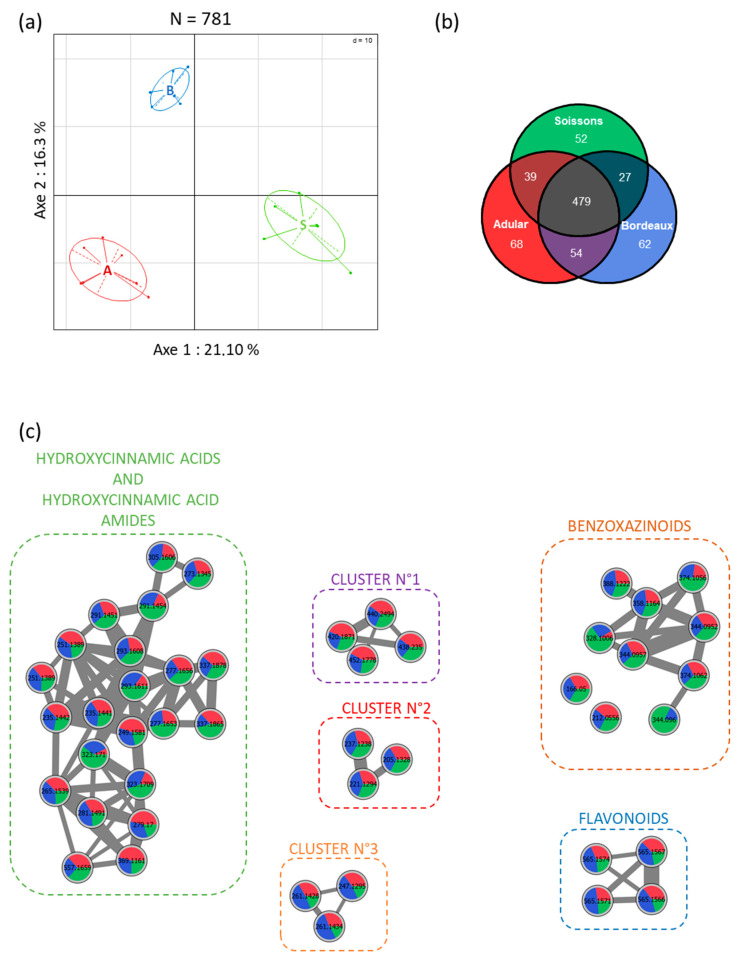
Principal component analysis obtained from LC-HRMS profiles of root extracts of the three wheat genotypes. Secondary metabolomes of wheat roots are displayed following PC1 = 21.1% and PC2 = 16.3% (n = 21 samples; 781 molecular ions) (**a**). The Venn diagram represents the significantly overproduced wheat metabolite ions according to the wheat genotypes (*p* ≤ 0.05; a Kruskal-Wallis nonparametric test corrected for false discovery; n=7 replicates) (**b**). Generation of a molecular network from MS/MS analyses of methanol extract of Adular, Bordeaux and Soissons wheat genotypes. Node identification corresponds to the masses of parent ions [M+H] and the pie charts show the production of metabolites according to the wheat genotypes. In order to enhance visibility, only clusters with more than three nodes were kept on the molecular network (**c**).

To examine the impact of genotypes on the wheat root metabolome, we profiled methanol root extracts by UHPLC-Q-TOF mass spectrometry. This untargeted analysis allowed the identification, after data filtration, of a total of 781 metabolite ions for all genotypes. An unsupervised principal component analysis of all metabolite ions revealed that wheat genotypes had major impacts on the root metabolome. The three wheat genotypes can be separated by the first two axes of the principal component analysis (Figure 1a), showing that they are able to produce different secondary metabolites or identical metabolites in different proportions at a sampling time of 21 days. Component 1, explaining 21.1% of the variation in the dataset, separated the Adular (A) and Soissons (S) genotypes. Component 2, which explains 16.3% of the variation in the dataset, separated the Bordeaux (B) samples from those of the other two genotypes. Univariate statistical analysis on ion abundances was used to identify metabolite ions that significantly differed between samples (Figure 1b). Among the 781 metabolite ions detected in the wheat genotypes extracts, 479 were produced in the same proportions in all wheat genotypes, while 302 were differentially produced between genotypes (Figure 1b). Most of these metabolite ions were produced by all genotypes, but in different proportions. The diversity of metabolites was approximately the same for all genotypes, and each genotype overproduced at least 50 specific metabolite ions. Nevertheless, the extracts from the Adular and Bordeaux samples shared the greatest number of metabolites, showing that the metabolomes of these two genotypes were close to each other.

Metabolite ions from wheat roots were identified using a molecular networking approach (Figure 1c, Table S1), which can cluster compounds belonging to the same chemical family according to their MS/MS fragmentation. The most represented chemical group was hydroxycinnamic acid derivatives, with 23 compounds annotated (Figure 1c). Most of the compounds were hydroxycinnamic acid amides, namely feruloyl or coumaroyl linked to an aliphatic amine such as putrescine, agmatine or cadaverine (Table S1). Another major class of wheat secondary metabolites was represented by the benzoxazinoid derivatives. Thirteen benzoxazinoid derivatives were detected in the chemical extracts. Some of them were identified as HBOA, DIMBOA, DHBOA, DIBOA, HMBOA, HDMBOA glycosylated or not, and their degradation product MBOA (Figure 1c, Table S1). Four flavonoid derivatives were also found in wheat extracts, as well as amino acids like tyrosine, tryptophan or methyl-proline (Figure 1c, Table S1). The majority of these compounds were produced by all wheat genotypes in different proportions, and a single benzoxazinoid derivative was produced only by the Soissons and Bordeaux genotypes (Figure 1c, BX4 in Table S1).

### 2.2. Pseudomonas Secondary Metabolism Modifications in Response to Wheat Root Extracts

The wheat root metabolites, harvested after 21 days of culture of the three genotypes, were put in contact with five rhizosphere-colonizing *Pseudomonas* strains by adding the extracts to MM medium at very low concentrations (i.e., 50 µg/mL and 25 µg/mL). Culturing *Pseudomonas* strains in minimal medium supplemented with root extracts at 50 µg/mL without any other carbon source did not support any bacterial growth (Figure S1a). However, when the same experiment was done in the presence of a carbon source (i.e., fructose, MMF medium), a significant increase of the absorbance at 600 nm was observed (Figure S1). Thus, any bacterial response to wheat root metabolites under these conditions could not be attributed to a trophic interaction, but rather to a signaling effect.

**Figure 2:**
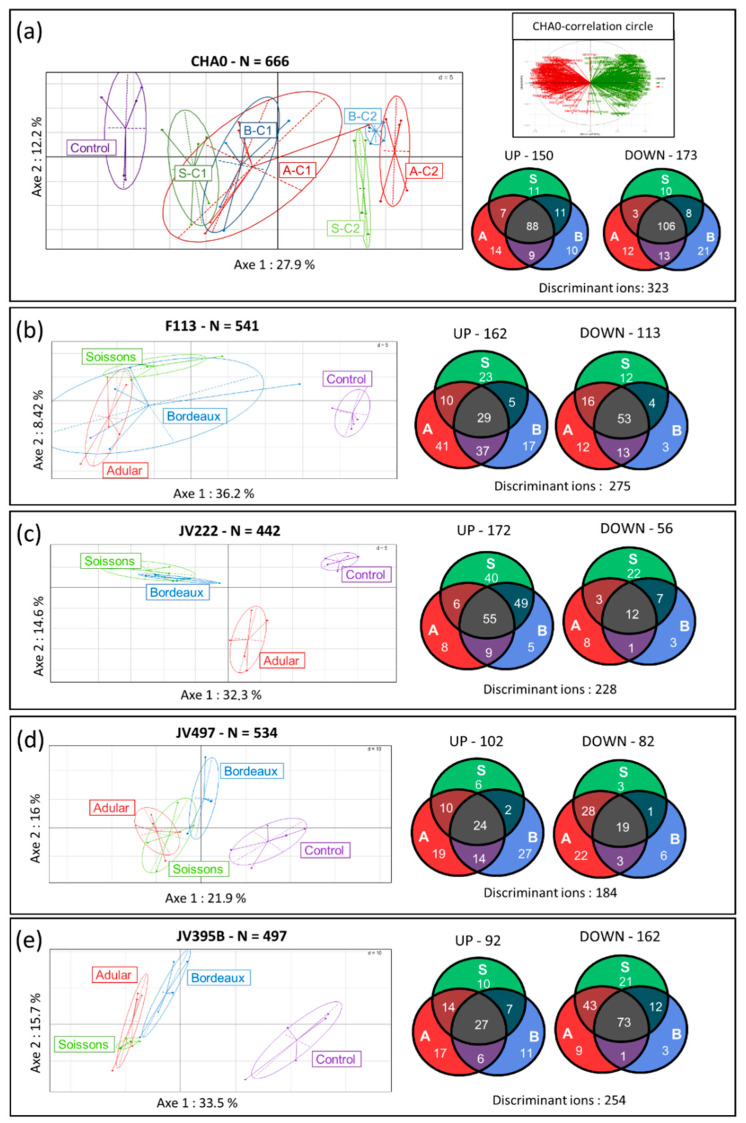
Principal component analysis obtained from LC−HRMS profiles of *Pseudomonas* strains. The LC-HRMS profiles are those of *Pseudomonas protegens* CHA0 (**a**), *Pseudomonas kilonensis* F113 (**b**), *Pseudomonas koreensis* JV222 (**c**), *Pseudomonas chlororaphis* JV497 (**d**) and JV395B (**e**) cultivated with wheat root extracts. All principal component analyses were associated with Venn diagrams representing proportions of metabolite ions of *Pseudomonas* significantly over and less produced in the presence of root extracts of Adular (A), Soissons (S) or Bordeaux (B) genotypes against control (*p* ≤ 0.05; a Wilcoxon nonparametric test corrected for false discovery; n=5 replicates). The principal component analysis obtained from the extracts of CHA0 under the influence of wheat extract at 25 (C1) and 50 µg/mL (C2) highlighted the concentration-dependent effects of the wheat extracts (**a**). The correlation circle associated with the principal component analysis for the CHA0 strain displays the metabolite ions involved in the separation of samples (**a**).

Then, the influence of root extracts at 50 and 25 µg/mL on the secondary metabolome of *Pseudomonas* was analyzed. Supernatants of *Pseudomonas* cultures were extracted by ethyl acetate and analyzed by UHPLC-Q-TOF mass spectrometry. This untargeted analysis identified 666 metabolite ions in the culture of *P. protegens* CHA0, 541 in that of *P. kilonensis* F113, 442 in that of *P. koreensis* JV222, 534 in that of *P. chlororaphis* JV497 and 497 in the culture of *P. chlororaphis* JV395B (Figure 2). A principal component analysis of all metabolite ions from every strain showed that the replicates of all conditions were well clustered; the control samples were separated from other conditions along the first axis, which represents 27.9%, 36.2%, 32.3%, 21.9% and 33.5% of variations in the supernatants of CHA0, F113, JV222, JV497 and JV395B, respectively (Figure 2). Furthermore, the addition of wheat extracts at 50 µg/mL (C2) led to more profound metabolic changes than the treatment at 25 µg/mL (C1), highlighting a concentration-dependent effect (Figure 2a; Figure S2). Finally, a principal component analysis carried out on all the metabolite ions generated from the supernatants of the JV222 strain allowed us to separate the JV222 metabolome under the influence of the Adular genotype from the other modalities along the second axis, which explained 14.6% of the variability (Figure 2c; Figure S2b).

The separation of bacterial samples under the influence of plant extracts from control conditions may signify that wheat metabolites triggered the production of new bacterial compounds or modulated the production of bacterial metabolites in *Pseudomonas* strains. The metabolite ions of *Pseudomonas* whose production was modified in the presence of root extracts were represented on the correlation circles associated to the principal component analyses (Figure 2). Statistical univariate analysis on all these metabolite ions showed that the production of numerous compounds was significantly altered (*p* ≤ 0.05) in response to root extracts from the three genotypes when compared to the control condition, for every tested strain (Figure 2). For *P. protegens* CHA0, a total of 323 metabolite ions representing 48.4% of the metabolome were significantly altered in response to at least one wheat extract with approximately equal numbers of metabolites, which were more or less produced compared to the control. The metabolome of the other strains, *P. kilonensis* F113, *P. koreensis* JV222, *P. chlororaphis* JV395B and *P. chlororaphis* JV497 was also impacted with 50.8%, 51.5%, 51.1% and 34.5%, respectively, of their metabolite ions significantly altered in the presence of root extracts (Figure 2). The impact of root extracts was not identical on all strains: JV395B produced most of its metabolites in lower amounts in response to wheat extracts (Figure 2e), while JV222 overproduced most of them (Figure 2c). Moreover, extracts from different genotypes altered the production of a different number of bacterial metabolite ions in each strain. In CHA0 cultures, the majority of metabolites were altered by the three wheat extracts, but the Bordeaux genotype led to a lower production of the largest number of bacterial compounds (Figure 2a). In contrast, F113 produced most of its metabolites in higher amounts only under the Adular extract, while JV222 was mostly influenced by the Soissons extract. However, comparison of the abundance of metabolite under the influence of wheat genotypes (i.e., Adular vs. Soissons; Adular vs. Bordeaux and Bordeaux vs. Soissons) did not show any significant differences except for strain JV222, and for only two metabolites produced by JV395B. This suggests that, for strains CHA0, F113, JV395B and JV497, the three wheat genotypes induced similar response trends but with different intensities. In contrast, 26.4% of the JV222 metabolite ions were differentially altered in presence of Soissons and Adular genotypes (Figure 2c). In short, root extracts induced important metabolic changes in all studied *Pseudomonas* strains, with specificities according to bacterial strains and wheat extracts.

### 2.3. Focus on Secondary Metabolites Involved in Plant-Bacteria and Bacteria-Bacteria Interactions

**Figure 3:**
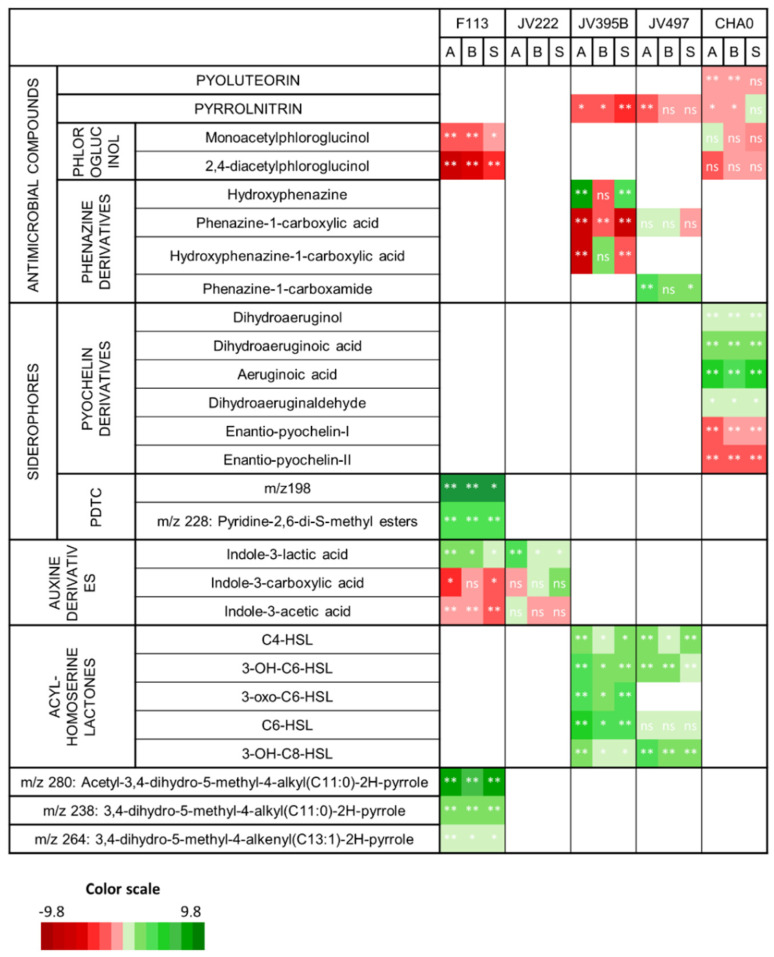
Influence of wheat root extracts on bacterial secondary metabolites involved in plant–bacteria or bacteria-bacteria interactions [28]. Heatmap projections represent Log2 fold changes in relative intensities of metabolites of *Pseudomonas* strains cultivated with root extracts from different wheat genotypes (Adular: A, Bordeaux: B or Soissons: S) and control. Statistically significant values against control (*p* ≤ 0.05; a Wilcoxon nonparametric test corrected for false discovery; n=5 replicates) were represented by: *: *p* ≤ 0.05 or ** *p* ≤ 0.01. The green color indicates metabolites with a greater intensity in the presence of root extracts, while the red color indicates metabolites with a greater abundance in the absence of root extracts. The white color means that the corresponding metabolite was absent from the bacterial extract.

Many of the secondary metabolites whose production appears to have been altered in response to wheat root extracts are involved in plant-bacteria and/or bacteria-bacteria interactions (Figure 3).

**Figure 4:**
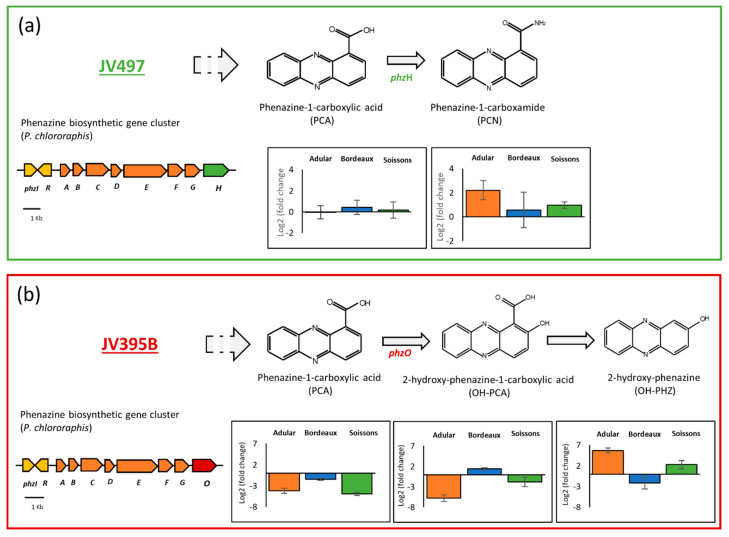
Plant metabolites interfere with the biosynthesis of phenazine-1-carboxylic (PCA) derivatives. The figure displays, for *Pseudomonas chlororaphis* JV497 (**a**) and JV395B (**b**), the phenazine biosynthetic pathway (adapted from Chin-A-Woeng et al. (2003) [29]), the organization of the phenazine biosynthetic operon in *Pseudomonas chlororaphis* JV497 (**a**) and JV395B (**b**), and the relative intensities (Log2 fold changes) of phenazine derivatives of *Pseudomonas* cultivated in the presence of wheat root extracts compared to the control condition (culture in the absence of root extracts) (n = 5 replicates).

*Pseudomonas* strains are able to produce a large panel of antimicrobial compounds (i.e., pyoluterorin, pyrrolnitrin, 2,4-diacetylphloroglucinol and phenazines). Pyoluteorin, pyrrolnitrin and phloroglucinols were either not impacted or were less produced in response to wheat extracts (Figure 3). Some differences can be highlighted according to the producing strains. For example, DAPG was significantly less produced by F113 while its production was not significantly impacted in CHA0. It is nevertheless relevant to note that, in our culture conditions, the production of DAPG was lower in CHA0 compared to F113, even when the latter was grown in the presence of root extracts. The production of pyrrolnitrin was fourfold reduced in JV395, while not significantly impacted in CHA0 (Figure 3). Lastly, phenazines, produced by the *P. chlororaphis* strains, did not display the same behavior as the other antimicrobial compounds (Figure 3). Indeed, the production of phenazine derivatives originating from a similar biosynthetic pathway was not altered in the same way in response to root extracts. In *P. chlororaphis* JV395B, the first phenazine derivative (i.e., phenazine-1-carboxylic acid, PCA) was significantly less produced in the presence of wheat extracts, while other phenazine derivatives (produced via the monooxygenase PhzO) were more accumulated (Figure 4b). Nevertheless, significant differences according to wheat genotypes were observed. Indeed, the Adular and Soissons extracts led to a higher production of the last derivatives of the pathway (i.e., hydroxyphenazine (OH-PHZ)), while the intermediate (i.e., hydroxyphenazine-1-carboxylic acid, OH-PCA) was more produced only in response to the Bordeaux extract. *P. chlororaphis* JV497 also responded to wheat extracts by producing the last derivative of this biosynthesis pathway, phenazine-1-carboxamide (PCN), in a higher amount (Figure 4a).

**Figure 5:**
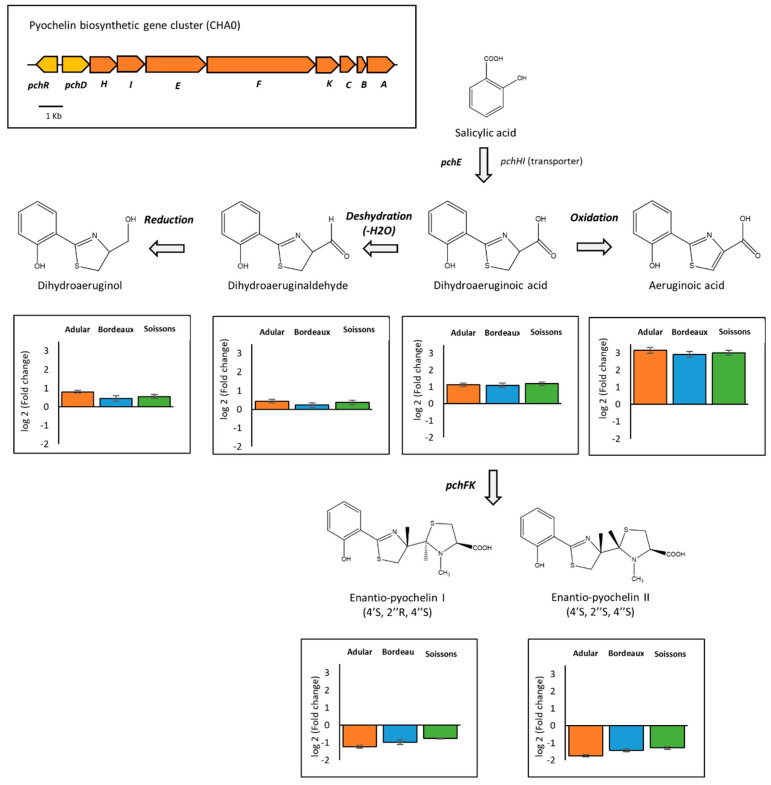
Plant metabolites interfere with the biosynthesis of pyochelin. The figure shows the pyochelin biosynthetic pathway (adapted from Ye et al. 2014 [30]), the organization of the pyochelin biosynthetic operon in *Pseudomonas protegens* CHA0, and the relative intensities (Log2 fold changes) of metabolites of *Pseudomonas* cultivated in the presence of wheat root extracts compared to the control condition (culture in the absence of root extracts) (n = 5 replicates).

Regarding the biosynthesis pathway of enantio-pyochelins found exclusively in *P. protegens* CHA0 (Figure 5), the immediate precursor of enantio-pyochelin and its derivatives (i.e., dihydroaeruginoic acid, aeruginoic acid, dihydroaeruginol and dihydroaeruginaldehyde) (Figure S3) were more produced in response to wheat extracts, while both enantio-pyochelins I and II were less produced. Finally, auxin derivatives produced by *P. kilonensis* F113 and *P. koreensis* JV222 were also differentially modulated by root extracts. Indole-3-lactic acid was more produced by both strains in response to wheat extracts, whereas indole-3-acetic acid was less accumulated by F113 and not impacted in JV222. Interestingly, the production of indole-3-carboxylic acid and indole-3-lactic acid by JV222 was not impacted in the same way, depending on the wheat genotype used to obtain root extracts. The Adular extract led to an increased amount of indole-3-lactic acid, whereas the Soissons extract triggered the accumulation of indole-3-carboxylic acid (Figure 3).

In addition, all quorum-sensing mediators belonging to the N-acyl-homoserine lactone (AHL) family were overproduced by both *P. chlororaphis* strains in response to wheat extracts of all genotypes. For example, the amount of 3-OH-C_6_-HSL was increased by approximately three times in response to wheat extracts. Only C_6_-HSL production by JV497 was not significantly impacted (Figure 3).

Moreover, apart from these bacterial secondary metabolites known to be involved in plant–bacteria interactions, two newly described families of secondary metabolites were significantly accumulated in F113 in response to root extracts. On one hand, the production of two pyridine-2,6-thiocarboxylic derivatives (PDTC) *m/z* 198 and *m/z* 228 [28] was increased in response to plant extracts; for example, the *m/z* 198 compound was 930-, 927- and 358-fold overproduced in the presence of Adular, Bordeaux and Soissons extracts, respectively (Figure 3). On the other hand, the production of three atypical compounds described as acyl-dihydro-methyl-pyrrol derivatives [28], was also strongly influenced by root extracts. The derivative whose synthesis was mostly affected was the *m/z* 280 compound, with a 46-, 29- and 44-fold overproduction in the presence of Adular, Bordeaux and Soissons extracts, respectively (Figure 3).

## 3. Discussion

Wheat roots produce a large diversity of primary and secondary metabolites that can interact with the rhizomicrobiota. Plant can shape root microbiota and interfere with bacterial physiology [6,8,18]. Previous studies have investigated the impact of primary metabolites or purified secondary metabolites on bacterial gene expression [31,32,33]. In our study, through a cross plant–bacteria metabolomics approach, we evaluated the impact of secondary metabolites from three wheat genotypes, harvested at one time point during the vegetative growth (i.e., 21 days), on the production of secondary metabolites by five rhizobacterial *Pseudomonas* strains.

### 3.1. Wheat Root Metabolites Differ Depending on Plant Genotypes

A total of 781 metabolite ions were detected in root extracts from the three wheat genotypes (Figure 1). Among these, 61.3% were common and produced in identical proportions in all genotypes. The differences between genotypes mainly relate to the quantitative variations of common compounds. Only a few compounds were specific to a genotype, such as the BX4 benzoxazinoid detected only in the Soissons and Bordeaux genotypes (Table S1). Several plant metabolites can be clustered on the molecular network, indicating that they share closely related chemical structures, and thus belong to the same molecular families (Figure 1c), [34,35]. Most of them are phenolic compounds already described as the most-represented class of compounds in wheat [36].

The main cluster gathered 23 metabolites belonging to hydroxycinnamic acid (HCAs) and hydroxy cinnamic acid amides (HCAAs) (Figure 1c). HCAs and HCAAs belong to an important class of phenylpropanoid metabolites, HCAs being the most studied. They can be released in the rhizosphere and have biological activity on rhizobacteria. For example, it has been demonstrated that HCAs are able to regulate the virulence of *Agrobacterium tumefaciens* on tomato [37]. HCAAs are found in plant seeds and roots, where they are involved in plant defense, cell division, senescence or stress response [38,39]. Accumulation of some HCAAs in response to PGPR inoculation has also recently been described in rice roots [40]. Flavonoids such as apigenin derivatives (i.e., schaftoside [36]) were also detected in wheat root extracts (Figure 1c). These secondary metabolites have been shown to be important bioactive secondary metabolites in the rhizosphere [41]. Next to their pivotal role in the establishment of the *Rhizobia*–Leguminous plant symbiosis and in the initiation of tumorigenesis by *Agrobacterium tumefaciens* [42], some flavonoids may also play a role in association between cereals and PGPR [43].

Another cluster grouped 13 compounds belonging to the benzoxazinoid family (Figure 1c). Benzoxazinoids constitute an important class of specialized metabolites in *Poaceae* [44,45]. They are particularly produced in young seedlings [46] and associated with plant defense against insects or microbial pathogens [47]. Stable benzoxazinoid glucosides are stored in root cell vacuoles and can be actively exuded from plant tissue into the soil [48]. Once in the rhizosphere, benzoxazinoid glucosides come into contact with β-glucosidases and are hydrolyzed to reactive aglucones, which can exert direct effects on soil microorganisms [48]. In our study, by analyzing root extracts instead of root exudates, we detected more benzoxazinoid glucosides than aglucone forms. Moreover, the relative quantification demonstrated a contrasted accumulation according to wheat genotypes (Table S1), revealing that the three genotypes displayed a differential benzoxazinoid fingerprint. Benzoxazinoids can exert different biological activities on rhizosphere micro-organisms [15,49]. For example, DIMBOA can promote recruitment of the plant-beneficial bacteria *Pseudomonas putida* KT2440 onto maize roots, and by contrast, exert antimicrobial activity on the bacterial pathogen *Agrobacterium tumefaciens* [49]. Moreover, a recent study, performed on a benzoxazinoids-deficient maize mutant showed that benzoxazinoids can shape the rhizosphere microbiota and drive plant-soil feedbacks on growth and defense [15]. Benzoxazinoids can be degraded into different degradation products. The first is the MBOA, which is described as one of the more active form of benzoxazinoids [10,15,44], and was shown to have a major role in shaping the maize root microbiota [15]. Subsequently, MBOA can be transformed by different degradation pathways. A plant-detoxification mechanism leads to the formation of BOA-6-O-glucoside and BOA-*N*-glucoside, which is rapidly rearranged into glucoside carbamate that is non-toxic for micro-organisms [50]. MBOA degradation by bacteria and fungi can lead to the formation of 2-aminophenol (2-AP), which can subsequently dimerise into the stable and more bioactive compound 2-amino-3H-phenoxazin-3-one (APO) [51]. In our study, MBOA was detected in identical amounts in root extracts of the three genotypes, while other degradation products were not detected.

Finally, besides an important number of well-known secondary metabolites, the majority of secondary metabolites extracted from wheat roots remain to be annotated (Figure 1).

### 3.2. Plant Root Compounds Affect the Biosynthesis of Bacterial Secondary Metabolites via a Signaling Effect

The impact of root extracts from all genotypes (i.e., Adular, Bordeaux and Soissons) was tested at a low concentration (50 µg/mL) on five fluorescent *Pseudomonas* strains able to colonize the wheat root system. We demonstrated that all five strains were unable to use wheat extracts as a trophic source, as no growth was observed in the absence of fructose (Figure S1a). However, the addition of these extracts to the MMF medium triggered physiological modifications of all strains, as shown by the enhancement of OD_600_ absorbance (Figure S1). These results suggest that wheat extracts contain bioactive secondary metabolites and/or primary metabolites in concentrations too small to sustain bacterial growth. Conversely, the bacterial response observed in a fructose-complemented medium suggested a signaling activity of wheat extracts on the five *Pseudomonas* strains, as confirmed by the analysis of bacterial metabolomes. Indeed, a principal component analysis performed on bacterial metabolomes showed that each condition was separated from the control (Figure 2). Moreover, a concentration-dependent effect was highlighted due to the use of two concentrations of root extracts (25 µg/mL and 50 µg/mL) (Figure 2a, Figure S2). As shown by the multivariate analysis that separates all conditions along axis 1, the presence of plant extracts was the most important factor explaining the variation of the data. The biological elicitation of secondary metabolism has been reported in Actinobacteria [52]. Here, a similar result was observed with plant extracts from different wheat genotypes on the metabolome of *Pseudomonas*. For example, the signaling effect of the Adular extract on *P. kilonensis* F113 enhances the production of 36.3% of its excreted secondary metabolites (Figure 2b). Interestingly, all strains do not respond similarly to root extracts: the addition of root extracts increased the production of 75.4% of discriminant metabolites in *P. koreensis* JV222, whereas the synthesis of 63.8% of metabolites was reduced in *P. chlororaphis* JV395B (Figure 2).

Except for JV222 (discussed below), the influence of root extracts from all three genotypes on each strain was observed on the first axis of the multivariate analysis, and this segregation was due to identical variables on the corresponding correlation circles (Figure 2a). This suggests that extracts from all genotypes impacted similar bacterial metabolites. Venn diagrams (Figure 2) show the differential numbers of bacterial metabolites whose relative quantity differed depending on the influence of the wheat genotype; these differential numbers may be explained by discrepancies in the composition of the root extracts, in particular at the quantitative level. This cross-metabolomic approach highlighted a very complex and tricky regulation of the bacterial secondary metabolism of *Pseudomonas*. For *P. koreensis* JV222, a contrasted response was observed according to wheat genotypes, with extracts from Adular and Soissons differentially impacting the synthesis of metabolites (Figure 2c). This specific behavior of JV222 could be correlated to the phylogenetic distance between this strain and the other *Pseudomonas* strains used in this study [14]. Moreover, this strain is known to possess fewer biocontrol properties than the other studied strains that belong to the CPC (*P. corrugata/P. protegens/P. chlororaphis*) clade [14]. The differential activity of Soissons root extracts on JV222 could also be correlated with the phylogenetic distance between Soissons (belonging to halotype II) and the other genotypes (belonging to halotype X) [21].

### 3.3. Wheat Root Extracts Interfere with the Production of Bacterial Secondary Metabolites Involved in Biotic Interactions

In order to evaluate the impact of plant extracts on the expression of bacterial phytobeneficial properties, we focused our attention on bioactive secondary metabolites produced by the studied strains [14,28]. The secondary metabolism of the five *Pseudomonas* strains was previously described by us [28]. We have shown that *Pseudomonas* strains have the capacity to produce secondary metabolites involved in plant-bacteria or bacteria-bacteria interactions, as well as in their adaptation to the environment (Figure 3) [25,28,53]. Among them, the four strains *P kilonensis* F113, *P. protegens* CHA0 and *P. chlororaphis* JV395B and JV497 were shown to be able to produce antimicrobial compounds [28,53,54,55]. In contrast, the last strain, *P. koreensis* JV222, could be considered as a phytostimulator strain, as this bacterium is able to produce significant level of indole derivatives, including IAA [27]. IAA production is an important phytostimulatory property of rhizosphere bacteria due to its role in root development and modulation of plant gene expression [11,27].

The production of antimicrobial compounds was globally decreased in all *Pseudomonas* strains under the influence of root extracts (Figure 3). The influence of plants on the production of antimicrobial compounds has previously been investigated in CHA0 by using a fluorescent reporter [32,55,56]. First, the impact of 63 pure metabolites from plant and bacteria has been investigated on the in vitro expression of *phl* and *plt* operons (i.e., operons for the biosynthesis of DAPG and pyoluteorin), and most compounds could affect (positively or negatively) the level of DAPG and pyoluteorin [32]. Second, an in planta experiment on wheat showed that the expression of *phl* and *prn* (i.e., DAPG and pyrrolnitrin gene clusters) was induced by the plant, whereas expression of *plt* and *hcn* (i.e., pyoluteorin and HCN gene clusters) was not [56]. Finally, inoculation of CHA0 on barley infected or not with *Pythium* showed that *phlA* was upregulated only on infected plants [55]. These studies showed that the impact of the plant on antimicrobial production by CHA0 is dependent on the plant environment and the composition of root secondary metabolites. In our experiment, wheat genotypes were not infected by a fungal pathogen. We can suppose that wheat metabolites are not able to enhance the production of antimicrobial compounds as previously observed [55]. However, we should also consider that these compounds present signaling activity at sub-inhibitory concentrations [1,57]. For example, DAPG, when applied at low concentrations, regulates auxin production in plants [26], but also in the biostimulant strain *Azospirillum brasilense* Sp245 [58]. By lowering DAPG production, a plant might favor the signaling effect of DAPG.

The production of other compounds was impacted by root extracts with a complex modulation of several biosynthesis derivatives (Figure 3). The phenazine biosynthetic pathway is one of the most influenced by root extracts, leading to differential effects depending on the compounds (Figure 3, Figure 4). Phenazine derivatives, produced by *P. chlororaphis* strains JV395B and JV497, represent a large class of bacterial metabolites. Although phenazines are studied for their antimicrobial activity and their contribution to the protection of plants against pathogens [47], they also play important roles in bacterial physiology through their siderophore properties and their effect on biofilm production and root colonization [59,60,61]. *P. chlororaphis* JV395B and JV497 possess gene clusters for phenazine biosynthesis, allowing the synthesis of PCA. The two gene clusters only differ by the terminal gene, *phzH* for JV497 and *phzO* for JV395B [28]. *phzH* encodes a transaminase that catalyzes the transformation of PCA into PCN, while *phzO* encodes a monooxygenase leading to the production of hydroxylated derivatives of PCA (i.e., OH-PCA and OH-PHZ) [29] (Figure 4). In JV497, the amount of PCA was not modified in the presence of wheat extracts, while the quantity of PCN was increased in the presence of extracts from the Adular and Soissons genotypes (Figure 4a). This suggests that the phenazine gene cluster was upregulated, leading to the accumulation of the PCN derivative. In JV395B, PCA was less accumulated in the presence of extracts from all genotypes, while later derivatives of the biosynthetic pathway were overproduced. Indeed, the Bordeaux extracts triggered the accumulation of OH-PCA, while the Adular and Soissons extracts led to the overproduction of its decarboxylated derivative OH-PHZ (Figure 4b). A few studies have investigated the structure–activity relationship of phenazines. A more efficient conversion of PCA into OH-PCA was found to promote the attachment of *Pseudomonas* strains on surfaces and affect the architecture of mature biofilm [62]. Other authors suggest a role of extracellular DNA in biofilm structure under OH-PCA influence [63,64]. Moreover, our previous study regarding the metabolome of *Pseudomonas* showed that OH-PCA and OH-PHZ derivatives were strongly accumulated in the biofilm condition compared to the planktonic condition [28]. Nevertheless, in both *P. chlororaphis* strains, Bordeaux extracts induced fewer modifications on phenazine production than the other two extracts (Figure 4).

In *P. chlororaphis* strains, quorum sensing (QS) is known to be involved in the regulation of phenazine production due to at least two regulation systems, *phzI/phzR* and *aurI/aurR* [28,53,65]. The wide majority of AHL derivatives were overproduced by *P. chlororaphis* JV395B and JV497 strains in the presence of root extracts. AHLs are the most commonly described QS mediators in Gram-negative bacteria [66]. QS is a cell density-dependent system that occurs via the production and response to QS signals [67,68]. This system regulates a wide range of bacterial phenotypes like virulence, biofilm formation and production of secondary metabolites [67]. Plant metabolites can interfere with bacterial QS; for example, legume flavonoids increase AHL production in rhizobia, and consequently interfere with bacterial nodulation [1,69,70,71]. In *P. chlororaphis*, the positive impact of a plant on AHL synthesis may enhance root colonization through the increase of biofilm formation [72,73]. Moreover, overproduction of AHL may be related with modulation of phenazine. However, no AHL derivative was detected in F113, CHA0 or JV222 cultures [28], but QS regulation could also be mediated by signals other than AHLs. Actually, AHL receptors have also been discovered in bacteria unable to synthetize AHL, and some of these receptors are not only unable to respond to AHLs, but instead recognize some plant molecules and regulate the expression of bacterial genes involved in phytostimulatory or biocontrol properties [74]. Indeed, CHA0 possesses such a receptor, named PsoR, that specifically responds to wheat and rice (but not to cucumber) metabolites by activating the production of antimicrobial compounds, showing the key role of QS regulation in plant protection against pathogens [75].

### 3.4. Wheat Root Extracts Modify the Synthesis of Siderophore and Compounds Whose Role in Plant-Bacteria Interactions Remains to Be Investigated

The pyochelin biosynthesis pathway was also impacted by wheat root extracts (Figure 5). Pyochelin is an important bacterial siderophore, able to chelate iron and deliver it into bacterial cells. Pyochelin biosynthesis begins by the transformation, via the product of the *pchE* gene, of salicylic acid into dihydroaeruginoic acid, the latter then being transformed into enantio-pyochelines through PchF and PchK [30,76]. Moreover, dihydroaeruginoic acid can be transformed into other derivatives. Our untargeted metabolomic analysis detected enantio-pyochelins I and II, but also aeruginoic acid, dihydroaeruginoic acid, dihydroaeruginaldehyde and dihydroaeruginol (Figure 5; Figure S3). The production of these compounds was significantly modified in the presence of root extracts from all genotypes. Thus, the precursor dihydroaeruginoic acid and its derivatives (i.e., aeruginoic acid, dihydroaeruginaldehyde and dihydroaeruginol) were overproduced, while both enantio-pyochelins were less produced. Beside siderophore activity, pyochelin was also reported as a signaling compound that promotes systemic plant defense [77]. A comparison of the biological activity of pyochelin and its precursors remains to be undertaken. Nevertheless, it was suggested that aeruginaldehyde might be a new QS mediator in *P. aeruginosa* [78], but a more recent study refuted this hypothesis [79].

Another siderophore, PDTC, can also be produced by F113 [53]. In this study, we did not detect PDTC, which is known to be quite unstable in solution [80]. However, several PDTC derivatives (i.e., ester, thioester and acid derivatives of PDTC [81]) were detected in the culture supernatants of F113 (Figure 3), suggesting the potential ability of this strain to produce PDTC [28,82]. Additional experiments using other solvents (ethanol and ethyl acetate) for extraction confirmed that the two derivatives *m/z* 228 and *m/z* 198 were actually produced by F113, whereas the other derivatives (*m/z* 182, 196 and 212) resulted in artefacts due to degradation by methanol (data not shown). These two PDTC derivatives (*m/z* 228 and 198) were the compounds most impacted by the presence of wheat extracts (Figure 3). For example, the *m/z* 198 compound accumulated more than 900 times in the presence of root extracts from the Adular and Bordeaux genotypes. PDTC allows metal chelation in soil, and has been described in *Pseudomonas stutzeri* as useful in metal depollution of soil [83], but this compound has never been studied in the context of plant–microorganism interactions.

Other compounds accumulated in the presence of wheat root extracts were detected in the F113 supernatant (Figure 3); these compounds share quite similar mass spectra to compounds described as acetyl-3,4-dihydro-5-methyl-4-alkyl(C11:0)-2H-pyrrole, 3,4-dihydro-5-methyl-4-alkyl(C11:0)-2H-pyrrole and 3,4-dihydro-5-methyl-4-alkenyl(C13:1)-2H-pyrrole [28,84]. These compounds were also strongly overproduced in the biofilm lifestyle [28]. Thus, the modulation of their production by plant compounds suggests that they could be involved in plant–bacteria interaction.

### 3.5. Despite Limitations, Our Approach Has Successfully Evidenced Key Metabolites Involved in Wheat-Pseudomonas Interactions

The approach we adopted has been successful in evaluating the impact of wheat extracts on the plant-beneficial properties of *Pseudomonas,* and in identifying both plant and bacterial metabolites involved in the wheat–*Pseudomonas* interaction. Nevertheless, it is important to consider limitations of this approach compared to other techniques. We chose to cultivate wheat genotypes in non-sterile soil in order to be as close as possible to field conditions. Due to these culture conditions, the recovery of a sufficient amount of exudates in order to evaluate their biological activity on *Pseudomonas* strains would have been very challenging [85]. Moreover, the exudation process depends on different parameters like root architecture, salinity or pH, and exhibits significant variability between replicates [86,87]. Consequently, our choice fell on root extracts instead of root exudates. The limitation of this method is that the root extracts represent the entire metabolome of the root, instead of only the exuded compounds capable of interacting with rhizosphere bacteria. These difficulties could be avoided by growing wheat in a hydroponic gnotobiotic system, but this type of experiment is far removed from natural conditions and may lead to a root metabolite content that is significantly different from that of plants grown in the field [15,87]. It is also important to note that the annotated class of root metabolites such as flavonoids, HCA, HCAA or benzoxazinoids has also been detected in other works on cereal root exudates [15,44,86,87]. Another limitation of our work is that root extracts were harvested after 21 days of culture, so all the differences were observed only at one time point. It is likely that metabolite profiles evolve over time, and that the profiles of Adular and Bordeaux remain close over time compared to that of the Soissons genotype.

## 4. Materials and Methods

### 4.1. Bacterial Strains and Media

This study was performed on five strains belonging to the fluorescent *Pseudomonas* group: *Pseudomonas kilonensis* F113 isolated in 1992 from the sugar beet rhizosphere [23]; *Pseudomonas protegens* CHA0 from tobacco rhizosphere [22]; and three *Pseudomonas* strains isolated in our lab in 2013 [14] from bulk soil of maize fields, *Pseudomonas chlororaphis* JV395B and JV497, and *Pseudomonas koreensis* JV222. These five *Pseudomonas* strains have been shown to colonize roots of different wheat cultivars in the same way [28]. *Pseudomonas* strains were routinely grown in King’s B (KB) agar medium [88], Luria Bertani (LB) broth medium [89] at 28 °C and Minimum medium (MMF), which is composed of one carbon source fructose (14.4 g/L), NH_4_Cl (1 g/L), KH_2_PO_4_ (1.36 g/L)/K_2_HPO_4_ (1.74 g/L), MgSO_4_ (0.8 g/L) and a mix of 15 amino acids (alanine, arginine, asparagine, aspartic acid, cysteine, glutamic acid, glycine, histidine, isoleucine, leucine, phenylalanine, threonine, tryptophan, tyrosine and valine; at 0.01 mg/mL each).

### 4.2. Extraction of Metabolites from Wheat Roots

Three bread wheat (*Triticum aestivum* L.) genotypes, Bordeaux 113 (7973), Adular (797) and Soissons (6607) were used in this work (source: UMR GEDC, INRA Clermont-Ferrand). Accessions Bordeaux 113 and Adular belong to the same accession group (haplotype X) and the Soissons accession belongs to another group (haplotype II) [21]. Root metabolites were collected from plants grown in 2-dm^3^ jars containing non-sterile, sieved (Ø4 mm, sieve mesh size), loamy soil (16.2% clay, 43.9% silt and 39.9% sand, pH 7.0, in water; 2.1% organic matter [90]), collected from the surface horizon of a luvisol at an experimental farm in La Côte-St-André (France). Each jar contained three seeds, and seven jars were used for each treatment (n = 21). The jars were randomly placed in a glasshouse at 20°C with a 16:8 h day:night photoperiod (relative humidity 45% during the day and 65% at night). The soil was watered at a water content of 20% (*w/w*). At 21 d after planting, the plants were dug up and soil that did not adhere to roots was discarded. Each plant root system and adhering soil were dipped into liquid nitrogen to avoid enzymatic reactions and freeze-dried by lyophilisation (Alpha 1-4 LSC Christ, Osterode, Germany). Preliminary experiments under the same conditions were carried out in order to determine the conditions of wheat growth and to assess the reproducibility of the data (data not shown).

For the extraction of metabolites, three freeze-dried root systems of the same condition were pooled (leading to seven replicates per condition) in an Eppendorf tube, which was immediately soaked into liquid nitrogen. Roots were crushed using a ball mill (TissueLyser II; Qiagen, Courtaboeuf, France). Extraction was performed using 1.5 mL methanol for 150 mg of dry sample by shaking 10 min at 150 g and sonicating 10 min; samples were then subjected to centrifugation in order to recover the supernatant. The extraction was performed twice, and extracts were pooled and dried using a Speedvac-assisted evaporation (Labconco, Kansas City, MO, USA). Each sample was then re-suspended in methanol in order to obtain a concentration of 10 mg/mL. Preliminary experiments under the same conditions were made in order to develop analytical methods and to assess the reproducibility of the data (data not shown).

### 4.3. Evaluation of the Effect of Wheat Root Extracts on Growth of Pseudomonas and on the Production of Bacterial Secondary Metabolites

First, the influence of the root metabolites collected from wheat genotypes, Adular, Bordeaux and Soissons, was assessed on the growth of *P. kilonensis* F113, *P. protegens* CHA0, *P. chlororaphis* JV395B, *P. chlororaphis* JV497 and *P. koreensis* JV222, using the Bioscreen device (Labsystems, Helsinki, Finland) in MM medium supplemented or not with fructose (i.e., MMF and MM). Bacterial inoculants were obtained after an overnight growth in LB medium and two washes with MM broth. MM and MMF were supplemented with wheat extracts at 50 µg/mL (1% methanol). Methanol and uninoculated medium were used as controls, leading to 42 conditions. The experimentation was carried out in Bioscreen plates (2 × 100 wells). Lastly, 190 µL of MM and MMF were inoculated with 10 µl of preculture, leading to an initial population of 5.10^6^ CFU/mL in 200 µL of culture per well. Then, plates were incubated in the Bioscreen automate under agitation at 28 °C. Optical density (OD) at 595 nm of the cell suspensions was automatically read at regular intervals of 20 min over three days.

Thus, the influence of the root metabolites collected from the three wheat genotypes was assessed on the metabolome of the five *Pseudomonas* strains in MMF medium supplemented with wheat extracts at 25 µg/mL and 50 µg/mL (1% methanol) over a six-day period. Methanol and uninoculated medium were also used as control, leading to 42 conditions. All 42 conditions were performed in five replicates (n = 5), leading to a total of 210 samples. After six days, 200 µL cultures from each of 210 samples were transferred in Eppendorf^®^ (Hamburg, Germany) and extracted with 200 µL of ethyl acetate. Liquid/liquid partition was achieved by 15 min of agitation and recovery of the upper organic phase after a 5 min-rest. The extraction protocol was repeated once, giving a total volume of 400 µL per sample. After that, the organic phase (ethyl acetate) was dried using a SpeedVac (Labconco, Kansas City, MO, USA). Dried extracts were suspended in 30 µL of methanol and centrifuged for 5 min at 12000 g. Thirty microliters were then transferred into vials for LC-MS analysis. A quality-control (QC) sample was prepared by mixing 1 µL of each sample (210 samples) in order to control the analytical repeatability during the UHPLC-MS analysis. Preliminary experiments under the same conditions were performed in order to develop analytical methods and to assess the reproducibility of the data (data not shown).

### 4.4. LC-HRMS Analysis

Wheat and bacterial extracts were analyzed on an Accurate-Mass Q-TOF LC-MS 6530 with an LC 1290 Infinity system (Agilent technologies^®^, Santa Clara, CA, United States). The separation was carried out at 40 °C using a 120 EC-C18 column (3.0 mm × 100 mm × 2.7 µm; Agilent Poroshell). Each sample (3 µL) was injected at the head of the column, and the column was eluted at 0.7 mL/min with a solvent gradient using solvent A (water with formic acid 0.4% (*v/v*)) and solvent B (acetonitrile). The gradient for wheat extracts was obtained by increasing the proportion of solvent B, step by step, from 1% to 15% during 8 min; 15% to 100% during 8.5 min, followed by a 4 min isocratic phase with 100% solvent B; back from 100% to 1% in 0.5 min and equilibration at 1% solvent B during 3 min, until the end of the run at 24 min. The gradient for bacterial extracts was carried out by increasing the proportion of solvent B from 10% to 36% during 4.5 min; 36% to 100% during 4 min, followed by a 2 min isocratic phase with 100% solvent B; back from 100% to 10% in 0.5 min and equilibration at 10% solvent B during 3 min, until the end of the run at 14 min.

Mass analyses were made in the positive mode, with the nebulization gas (nitrogen) at a flow of 10 L/min and 40 psg pressure. The capillary tension was 3000 V and gave an ionization energy of 100 eV. Mass spectra were recorded from m/z 50 to 1200. Complementary tandem mass spectrometry analyses (MS/MS) were performed thanks to a collision induced dissociation (CID) with a collision energy of 20 eV. Analyses included QC and blank samples every 10 sample runs. All chromatograms were explored with Mass Hunter Qualitative Analysis B.07.00 software (Agilent Technologies).

### 4.5. Data Processing and Statistical Analysis of Secondary Metabolites of Wheat and Pseudomonas

The metabolomic analysis workflow was focused on small molecules (<1000 Da) for both wheat root extracts and *Pseudomonas* metabolomes. LC-MS raw files were converted to mzXML format using MS convert “ProteoWizard 2.1” with filtering of m/z and eliminating those that were outside the range of 50–450 seconds for *Pseudomonas* extracts and 80–880 seconds for wheat root extracts [91]. The data were then processed using both the R software and the collaborative Galaxy platform “Workflow4metabolomics” version 3.3 [92]. Then the overall workflow of data processing was as follows: (1) peak extraction (xcms R package 3.6.1) [93]; (2) peak alignment (xcms R package 3.6.1); (3) elimination of ions also found in blank samples; (4) elimination of ions having a coefficient of variation (CV) of signal intensity higher than 25% in the QC pool sample; (5) elimination of the isotopic ions or adducts that represent the same molecule (CAMERA R-package and manual control validation) [94]; and finally (6) a last step of normalization was performed according to the total chromatogram intensity given the relative intensity for each pre-processed ion. Data-processing parameters are presented in Table S2 for both wheat and *Pseudomonas* samples. Statistical analyses were realized using R 3.5.1©2018 software version. The principal component analyses were made with R package Ade4 [95]. The nonparametric univariate statistical analysis was made using a nonparametric rank sum statistical test corrected for false discovery (*p* ≤ 0.05).

### 4.6. Molecular Network Analysis and Identification of Metabolite

The identification of wheat secondary metabolites was carried out using a molecular networking (MN) approach. MN was realized from MS/MS data after analyses of wheat root extract according to the protocol described by [96]. MS/MS data were converted to .mzXML format using MS convert “ProteoWizard 2.1” and were processed using MZmine 2 v2.53. All parameters are presented in supplementary data (Table S3). Molecular networking was realized thanks to the Metgem software with cosine score = 0.6 [35]. The molecular-networking approach allowed us to cluster compounds belonging to the same chemical family. Thus, the identification of tryptophan, phenylalanine and the benzoxazinoids DIBOA and MBOA was confirmed by comparison of the retention time and accurate mass with those of pure chemical standards (Table S4); other benzoxazinoid derivatives were characterized by comparing their UV spectra, accurate masses and MS/MS fragmentations to those of standards of DIBOA and MBOA. Benzoxazinoids MS/MS fragmentations were also compared to previous works from our group [83]. The hydroxycinnamic part of the HCA and HCAA derivatives were characterized by comparing their UV spectra, accurate masses and MS/MS fragmentations to those of standards of fumaric acid, caffeic acid and succinic acid (Table S4). HCAA derivatives were annotated due to neutral loss comparison with data available in Li et al. [97]. Finally, flavonoid derivatives were annotated as schaftoside/isoschaftoside according to their apigenin typical UV spectra and by comparing accurate masses and MS/MS fragmentations to the work of Dinelli et al. [36], which described phenolic compounds in different wheat varieties. *Pseudomonas* secondary metabolites were annotated according to the same type of protocols and annotation information was previously detailed in Rieusset et al. [28].

## 5. Conclusions

Secondary metabolites allow an organism to be adapted to a biotic and abiotic environment [98]. In this context, the differential production of *Pseudomonas* secondary metabolites reflects its physiological response to wheat root metabolites. The biological role of most of these metabolites is still unknown. Moreover, the production of some metabolites is interconnected; for example, the production of phenazine by *P. chlororaphis* strains is under the control of AHLs [65]. Nevertheless, an overview of the impact of wheat roots on the known bioactive metabolites of *Pseudomonas* suggests an enhancement of bacterial interactions. Indeed, on one hand, the biosynthesis of antimicrobial compounds was mainly not impacted. These were even less accumulated in the presence of root extracts, suggesting that healthy wheat does not promote *Pseudomonas* antagonist activities against other microbes and/or plant. On the other hand, several findings suggest that plant metabolites induce a signaling process in the five studied *Pseudomonas* strains, i.e., the low concentration of antimicrobials, the overproduction of signaling compounds like AHLs, and the overproduction of phenazine derivatives involved in biofilm architecture (i.e., OH-PCA) or of the pyochelin precursor presenting displaying a signaling activity (i.e., aeruginaldehyde derivatives) [28,62,63,78]. These physiological changes may impact interactions of *Pseudomonas* strains with wheat itself, as well as with other microorganisms within the rhizomicrobiota.

## Data Availability

The data presented in this study are available in www.mdpi.com/xxx/s1.

## Supplementary Materials

The following are available online at https://www.mdpi.com/2218-1989/11/2/84/s1, Figure S1: Effect of wheat root extracts (50 µg/mL) on the growth of Pseudomonas strains in minimum medium (MM) with or without fructose after 3 days, Figure S2: Principal component analysis obtained from LC−HRMS profiles of Pseudomonas kilonensis, Figure S3: MS/MS fragmentation spectra of enantio-pyochelin I and II, aeruginoic acid, dihydroaeruginoic acid, dihy-droaeruginol and dihydroaeruginaldehyde, Table S1: Chemical characterization of annotated secondary metabolites combined with statistically significant differences between wheat genotypes (Adular, Bordeaux, Soissons), Table S2: Setting parameters used for processing of the metabolomics data, Table S3: Molecular networking: MZmine 2 data-preprocessing parameters, Table S4: List of the 15 chemical standards used in UHPLC-DAD-qTOF analyses.
