# Supplementary material for: A Cross-Metabolomic Approach Shows that Wheat Interferes with Fluorescent Pseudomonas Physiology through Its Root Metabolites
Source: Metabolites. 2021 Jan 31;11(2):84. doi: 10.3390/metabo11020084 (PMC7911646; doi:10.3390/metabo11020084)
Supplement: Supplementary file 1 [file metabolites-11-00084-s001.pdf]

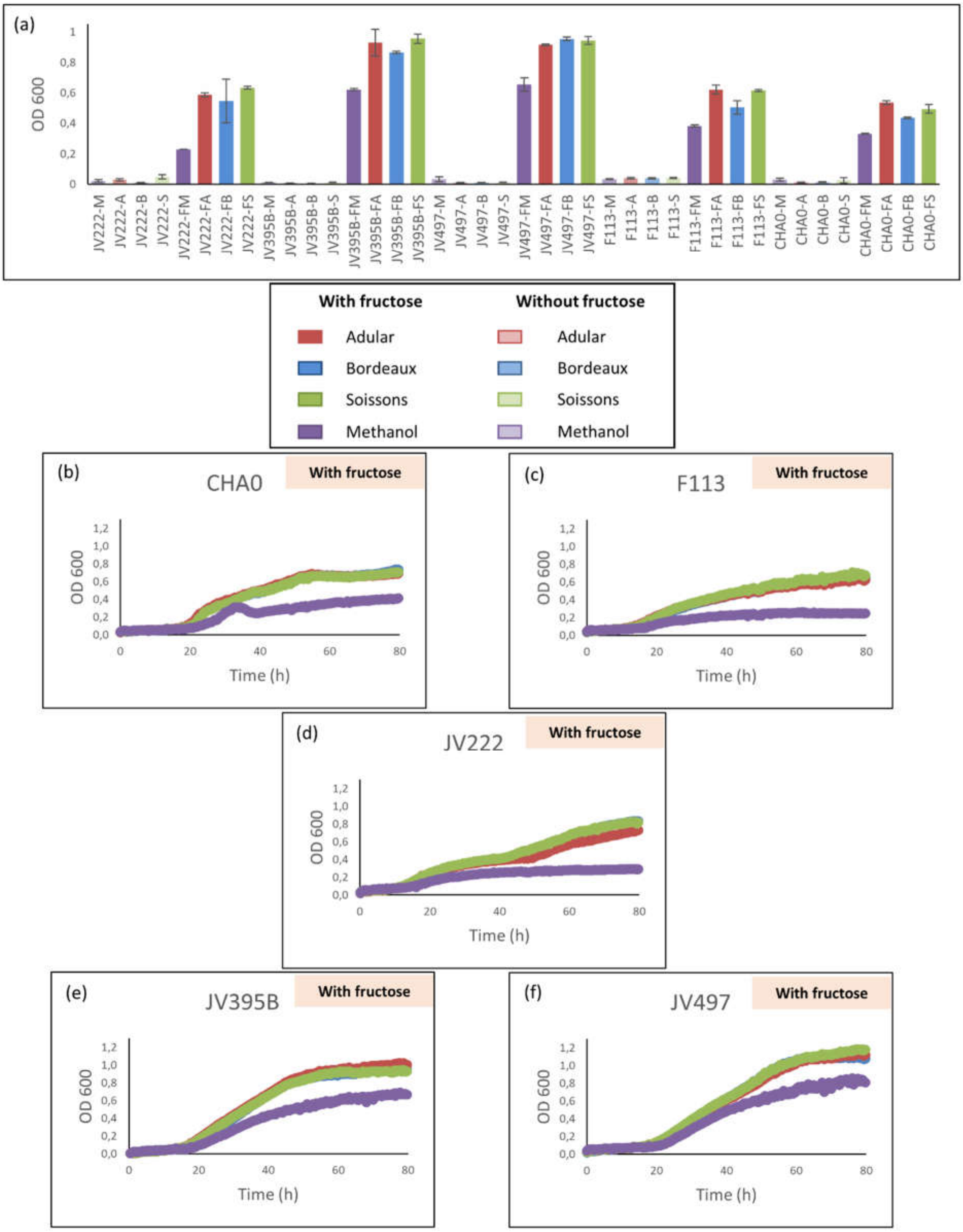

Figure S1. Effect of wheat root extracts (50 µg/mL) on the growth of *Pseudomonas* strains in minimum medium (MM) with or without fructose after 3 days (a). Growth curves of *Pseudomonas protegens* CHA0 (b), *P. kilonensis* F113 (c), *P. koreensis* JV222 (d), *P. chlororaphis* JV395B (e) and JV497 (f) in MM fructose complemented with wheat extracts from genotype Adular, Bordeaux and Soissons at 50 µg/mL.

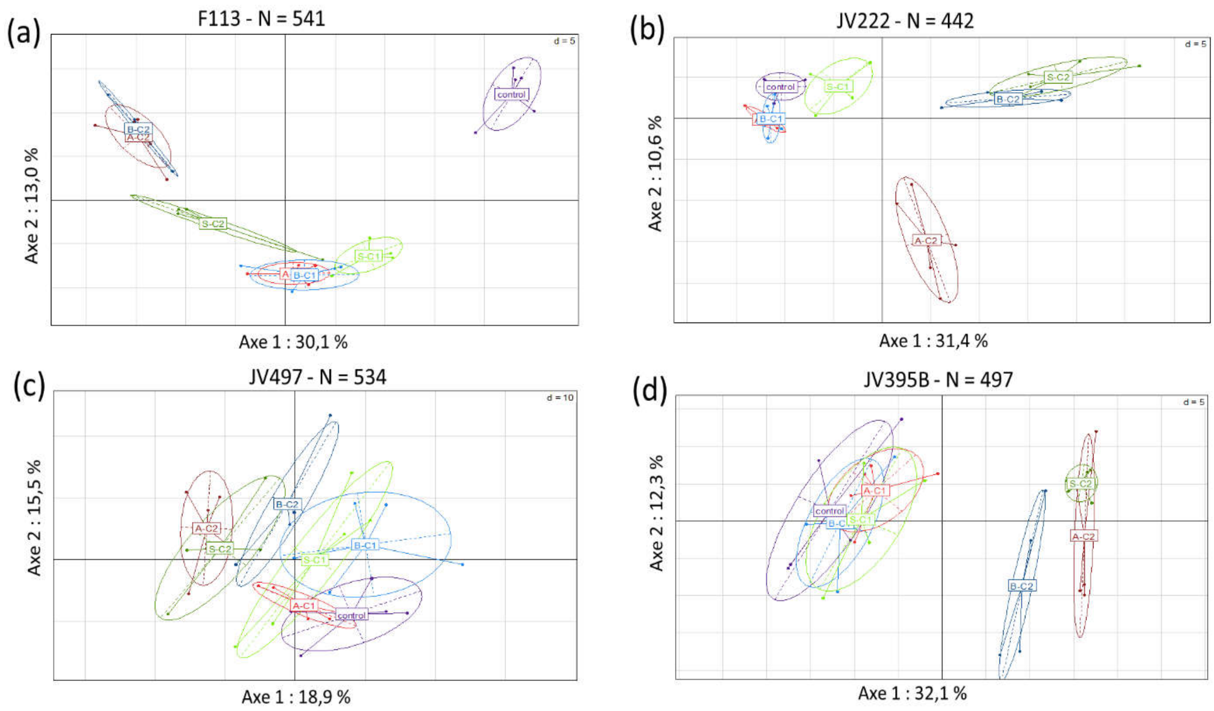

Figure S2. Principal component analysis obtained from LC−HRMS profiles of *Pseudomonas kilonensis* F113 (a), *P. koreensis* JV222 (b), *P. chlororaphis* JV497 (c) and JV395B (d) cultivated in MMF medium complemented with wheat root extract from Adular (A), Soissons (S), or Bordeaux (B) at 25 mg/mL (C1) and 50 µg/mL (C2) after 6 days incubation. Uninoculated medium was used as control.

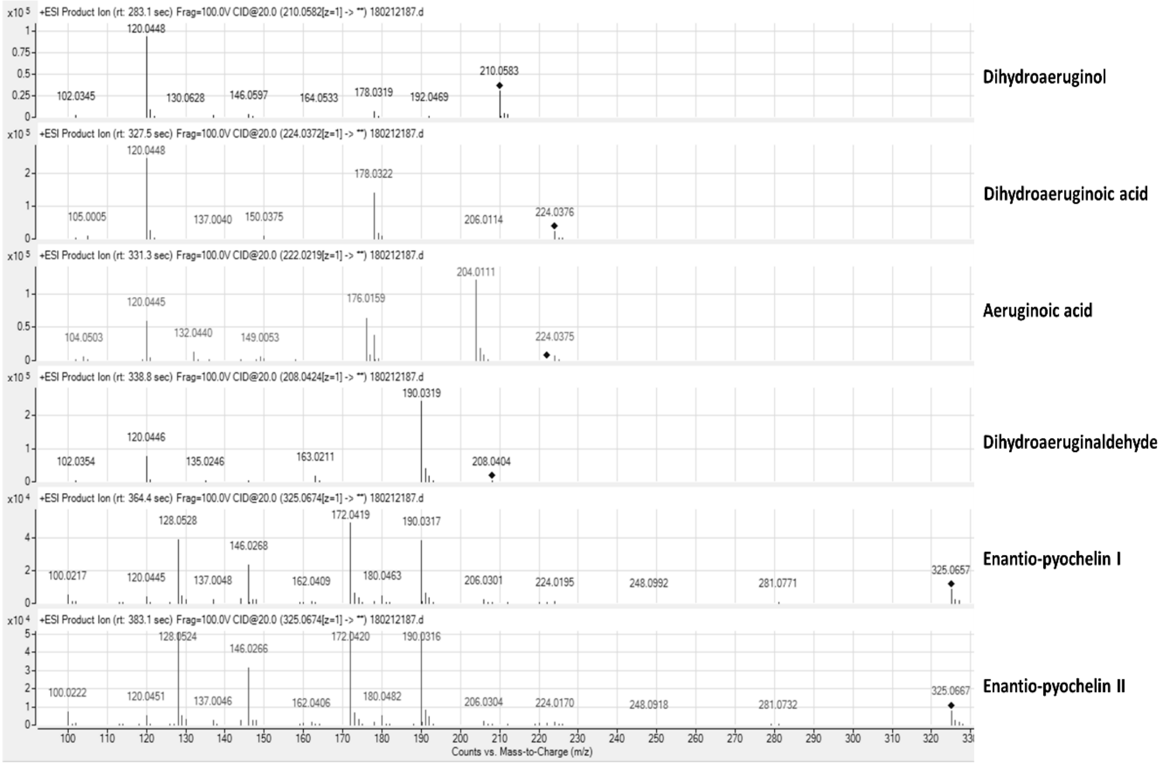

Figure S3. MS/MS fragmentation spectra of enantio-pyochelin I and II, aeruginoic acid, dihydroaeruginoic acid, dihydroaeruginol and dihydroaeruginaldehyde.

Table S1. Chemical characterization of annotated secondary metabolites combined with statistically significant differences between wheat genotypes (Adular, Bordeaux, Soissons). Data include compound number (N°), retention time (rt, min), UV maximum absorption (nm), molecular formula, negative high resolution mass spectrum (HR-MS (-)), positive high resolution mass spectrum (HR-MS (+)), MS/MS fragmentation in positive ionisation mode (HR-MS/MS (+)), neutral loss and putative annotation.

N°	rt (min)	UV (nm)	Formula	HR-MS (-)	HR-MS (+)	HR-MS/MS (+)	Neutral loss	Putative annotation	Adular*	Bordeaux*	Soissons*
**AMINO ACIDS AND DERIVATIVES**											
1	0.96	-	$C_6H_{11}NO_2$	129.0152	130.0861	-	-	Methyl proline	-	-	-
2	1.58	275	$C_9H_{11}NO_3$	180.0648	182.0568	136.0766; 123.0430; 119.0447	-	Tyrosine	a	a	a
3	4.83	218; 278	$C_{11}H_{12}N_2O_2$	203.0811	205.0974	146.0611; 132.0809; 118.0667	-	Tryptophan	a	a	a
4	3.29	-	$C_9H_{11}NO_2$	164.0697	166.0501	120.0809; 103.0538; 84.9605	-	Phenylalanine	a	a	a
**BENZOXAZINOIDS**											
5	3.88	264; 286	$C_{14}H_{17}NO_9$	342.0804	344.0957; 366.0786 [M+Na]	182.0439 ; 164.0333 ; 136.0383	162	DHBOA-Glc	b	c	a
6	4.09	264; 288	$C_{14}H_{17}NO_9$	342.0813	344.0952; 366.0786 [M+Na]	182.0437 ; 164.0333 ; 136.0412	162	DIBOA-Glc	ab	b	a
7	5.96	254; 280	$C_{14}H_{17}NO_8$	326.0858	328.1006; 350.0835 [M+Na]	166.0484 ; 148.0358 ; 120.0424	162	HBOA-Glc	c	b	a
8	6.03	255; 282	$C_{14}H_{17}NO_9$	342.0817	344.0960; 366.0787 [M+Na]	182.0450 ; 164.0314 ; 136.0351	162	BX4	c	b	a
9	6.20	265; 287	$C_{15}H_{19}NO_{10}$	372.0912	374.1062; 396.0907 [M+Na]	212.0550 ; 194.0444 ; 166.0488	162	BX5	b	b	a
10	6.39	267; 290	$C_{21}H_{29}NO_{15}$	534.1406	558.1490 [M+Na]	212.0550 ; 194.0444 ; 166.0488	162 X 2	DIMBOA-Glc-Glc	a	a	a
11	6.96	263; 286	$C_{15}H_{19}NO_9$	356.0948	358.1164; 380.0977 [M+Na]	196.0610 ; 178.0506 ; 150.0523	162	HMBOA-Glc	c	b	a
12	7.17	265; 292	$C_{15}H_{19}NO_{10}$	372.0893	374.1056; 396.0884 [M+Na]	212.0537 ; 194.0531 ; 166.0483	162	DIMBOA-Glc	a	a	a
13	7.61	265; 290	$C_9H_9NO_5$	210.0371	212.0556	194.0439; 166.0479; 110.0599; 95.0464	-	DIMBOA	a	a	a

14	9.18	264; 294	$C_{16}H_{21}NO_{10}$	386.0993 432.1107 [M + FA-H]$^-$	388.1222; 410.1042 [M+Na]	226.0703 ; 208.0588 ; 194.0433 ; 166.0484	162	HDMBOA-Glc	b	a	a
15	9.33	231; 287	$C_8H_7NO_3$	164.0350	166.0489	110.0596; 95.0466	-	MBOA	a	a	a
FLAVONOIDS											
16	8.12	223; 270; 333	nd	563.1401	565.1571	nd	nd	Schaftoside /isoschaftoside	b	a	b
17	8.52	223; 270; 333	nd	563.1402	565.1567	nd	nd	Schaftoside /isoschaftoside	b	a	c
18	8.62	223; 270; 333	nd	563.1368	565.1570	nd	nd	Schaftoside /isoschaftoside	ab	a	b
19	9.03	223; 270; 333	nd	563.1330	565.1574	nd	nd	Schaftoside /isoschaftoside	b	a	b
HYDROXYCINNAMIC ACIDS AND HYDROXYCINNAMIC ACID AMIDES DERIVATIVES											
20	4.03	nd	$C_{13}H_{18}N_2O_2$	233.1259	235.1444	176.07 ; 147.0437 ; 119.0509	88	Coumaroyl-putrescine	a	a	a
21	4.12	nd	nd	249.0876	251.1386	174.0535 ; 147.0449 ; 119.0506	104	Coumaroyl derivative	a	a	a
22	4.40	270 ; 295	nd	249.1222	251.1407	174.0535 ; 147.0449 ; 119.0506	104	Coumaroyl derivative	a	a	b
23	4.93	300	$C_{13}H_{18}N_2O_2$	233.1296	235.1448	176.07 ; 147.0437 ; 119.0509	88	Coumaroyl-putrescine	a	a	b
24	4.28	288; 304	nd	291.1457	293.1615	164.0662; 147.0439	146	Coumaroyl derivative	b	a	a
25	4.39	290; 310	nd	289.1278	291.1451	255.1237; 147.0442; 127.0973	144	Coumaroyl derivative	c	b	a
26	5.02	280; 308	nd	279.1305	281.1511	225.0751; 177.0537; 145.0286	104	Feruloyl derivative	b	a	c
27	5.04	290; 323	nd	321.1550	323.1724	247.1205; 177.0524; 145.0258	146	Feruloyl derivative	c	b	a
28	5.26	288; 304	nd	291.1427	293.1615	164.0662; 147.0439	146	Coumaroyl derivative	b	a	a

**29**	**5.26**	**265; 295**	**$C_{14}H_{20}N_4O_2$**	**275.1200**	**277.1648**	**218.1206 ; 147.0436 ; 119.0503**	**130**	**Coumaroyl-agmatine**	**a**	**a**	**a**
**30**	**5.36**	**290; 310**	**nd**	**289.1281**	**291.1451**	**255.1237; 147.0442; 127.0973**	**144**	**Coumaroyl deriva-tive**	**b**	**b**	**a**
**31**	**5.73**	**290; 320**	**$C_{14}H_{20}N_2O_3$**	**263.1389**	**265.1523**	**225.1061; 177.0535; 145.0264; 117.0312**	**88**	**Feruloyl-putrecine**	**a**	**a**	**b**
**32**	**5.92**	**nd**	**$C_{14}H_{20}N_2O_2$**	**nd**	**249.1581**	**175.0803; 147.0436; 119.0504**	**102**	**Coumaroyl-cadaver-ine**	**a**	**b**	**c**
**33**	**6.03**	**290; 323**	**nd**	**321.1521**	**323.1724**	**247.1205; 177.0524; 145.0258**	**146**	**Feruloyl derivative**	**b**	**a**	**a**
**34**	**6.07**	**nd**	**$C_{17}H_{20}O_9$**	**367.1008**	**369.1181**	**177.0521; 149.0514; 145.0249**	**192**	**Feruloyl-quinic**	**a**	**b**	**b**
**35**	**6.27**	**nd**	**$C_{16}H_{24}N_4O_4$**	**335.1670**	**337.1885**	**207.0641; 175.0375; 147.0463**	**130**	**Sinapoyl-agmatine**	**a**	**b**	**a**
**36**	**6.32**	**265; 295**	**$C_{14}H_{20}N_4O_2$**	**275.1491**	**277.1648**	**218.1206 ; 147.0436 ; 119.0503**	**130**	**Coumaroyl-agmatine**	**a**	**a**	**a**
**37**	**6.71**	**nd**	**$C_{15}H_{22}N_2O_3$**	**nd**	**279.1693**	**262.0273; 177.0528; 145.0284**	**102**	**Feruloyl-cadaverine**	**a**	**b**	**c**
**38**	**7.59**	**nd**	**$C_{16}H_{24}N_4O_4$**	**335.1657**	**337.1858**	**207.0641; 175.0375; 147.0463**	**130**	**Sinapoyl-agmatine**	**a**	**a**	**a**
**39**	**11.89**	**297; 328**	**nd**	**nd**	**557.1687**	**177.0541; 169.0462; 145.0268**	**nd**	**Ferruloyl derivative**	**a**	**b**	**a**
**40**	**11.26**	**240; 295; 328**	**nd**	**nd**	**515.1603**	**177.0557**	**nd**	**Feruloyl derivative**	**a**	**b**	**a**
**41**	**11.49**	**295; 325**	**nd**	**nd**	**445.1483**	**177.0545 ; 145.0282**	**nd**	**Feruloyl derivative**	**a**	**a**	**a**
**42**	**11.84**	**288; 328**	**nd**	**nd**	**343.1106**	**187.0384 ; 173.0720 ; 157.0896 ; 131.0469**	**nd**	**Cinamoyl derivative**	**a**	**a**	**a**
**CLUSTER N°1**											
**43**	**3.38**	**225; 278**	**nd**	**nd**	**452.1778**	**193.0611; 177.0544; 141.0565**	**nd**	**nd**	**a**	**b**	**b**
**44**	**6.78**	**257; 300**	**nd**	**nd**	**420.1854**	**193.0475; 163.0600; 145.0508**	**nd**	**nd**	**a**	**b**	**b**
**45**	**9.94**	**-**	**nd**	**nd**	**438.2353**	**223.0574 ; 145.0491; 127.0412**	**nd**	**nd**	**a**	**b**	**a**

46	10.36	257; 282; 318	nd	nd	440.2503	223.0594; 193.0516; 163.0600; 145.0493	nd	nd	a	b	b
CLUSTER N°2											
47	2.98	-	nd	219.1106	221.1294	176.0720; 148.06673; 130.0639	nd	nd	b	a	b
48	3.83	-	nd	235.1076	237.1238	176.0705; 148.0759; 130.0650	nd	nd	c	a	b
49	4.05	-	nd	nd	205.1328	160.0744; 142.0602; 132.0816	nd	nd	a	a	a
CLUSTER N°3											
50	2.85	-	nd	245.1121	247.1295	184.0978; 130.0474; 84.0429; 72.0820	nd	nd	b	a	c
51	4.69	-	nd	259.1309	261.1428	246.0746; 84.0457	nd	nd	b	a	c
52	5.03	-	nd	259.1272	261.1434	198.1123; 132.1024; 86.0986	nd	nd	b	a	c

* Different letters in a row indicate statistically significant differences between genotypes (non-parametric test corrected for false discovery p value ≤ 0.05).

Table S2. Setting parameters used for processing of the metabolomics data.

Step	Method	Parameters	Values wheat root samples	Values *Pseudomonas* strains culture samples
**Peak detection (*Peakpicking*)** [a]	centWave	ppm	10	10
		mzdiff	0.05	0.001
		prefilter	3, 5000	3, 1500
		snthresh	10	10
		peakwidth	4, 15	4, 15
		noise	10000	2000
**Peak grouping (*group*)** [a]	density	bw	4	2
		mzwid	0.25	0.25
		minfrac	0.2	0.2
**FillPeaks**	chrom			

[a] data processed with xcms R-package on collaborative Galaxy platform "Workflow4metabolomics" version 3.3.

Table S3. Molecular networking: MZmine 2 data-preprocessing parameters.

Steps	Methods	Parameters	Values
**Mass detection**		Noise level MS1	0
		Noise level MS2	0
**chromatogram builder**	ADAP	Minimum group size of scan	4
		Group intensity threshold	3000
		Minimum highest intensity	4000
		m/*z* tolerance	0.005 (20 ppm)
**Deconvolution**	ADAP Wavelets algorithm	S/N threshold	8
		Minimum feature height	4000
		Coefficient/area threshold	20
		Peak duration range	0.05 – 1 min
		T_R wavelet range	0.01 – 0.07 min
**MS2 scans paired**		*m*/*z* tolerance	0.02 Da
		t_R tolerance	0.3 min
**Isotopologue grouping**	Isotopic peak grouper algorithm	*m*/*z* tolerance	0.005 (20 ppm)
		t_R tolerance	0.2 min
**Filtering**	Feature list rows filter	Retention time range	1 – 14 min
		**Keep only peaks with MS2 scan**	
**Peak alignment**	Join aligner module	*m*/*z* tolerance	0.005 (20 ppm)
		Weight for *m*/*z*	2
		t_R tolerance	0.5 min
		Weight for t_R	1
**Gap filled**		*m*/*z* tolerance	0.005 (20 ppm)
		t_R tolerance	0.5 min

Table S4. List of the 15 chemical standards used in UHPLC-DAD-qTOF analyses.

N°	Name	Molecular formula	Origin	Purity
1	Monoacetylphloroglucinol	$C_8H_8O_4$	Cayman Chemical	≥98%
2	2,4-diacetylphloroglucinol	$C_{10}H_{10}O_5$	Toronto Research Chemicals Inc.	≥98%
3	Pyrrolnitrin	$C_{10}H_6Cl_2N_2O_2$	SIGMA Chemical Co.	≥98%
4	Phenazine-1-carboxylic acid	$C_{13}H_8N_2O_2$	Toronto Research Chemicals Inc.	≥98%
5	3-OH-C6-HSL	$C_{10}H_{17}NO_4$	SIGMA Chemical Co.	≥98%
6	Dimethyl 2,6-pyridinedicarboxylate	$C_9H_9NO_4$	SIGMA Chemical Co.	≥98%
7	Indole-acetic-acid	$C_{10}H_9NO_2$	SIGMA Chemical Co.	≥98%
8	Tryptophan	$C_{11}H_{12}N_2O_2$	SIGMA Chemical Co.	≥98%
9	Phenylalanin	$C_9H_{11}NO_2$	SIGMA Chemical Co.	≥98%
10	Fumaric acid	$C_4H_4O_4$	SIGMA Chemical Co.	≥98%
12	Caffeic acid	$C_9H_8O_4$	SIGMA Chemical Co.	≥98%
13	Succinic acid	$C_4H_6O_4$	SIGMA Chemical Co.	≥98%
14	DIBOA-Glc	$C_{14}H_{17}NO_9$	-	-
15	MBOA	$C_8H_7NO_3$	SIGMA Chemical Co.	≥98%
